# Design, Recombinant Fusion Expression and Biological Evaluation of Vasoactive Intestinal Peptide Analogue as Novel Antimicrobial Agent

**DOI:** 10.3390/molecules22111963

**Published:** 2017-11-14

**Authors:** Chunlan Xu, Yu Guo, Xiangjin Qiao, Xiaoya Shang, Weining Niu, Mingliang Jin

**Affiliations:** The Key Laboratory for Space Bioscience and Biotechnology, School of Life Sciences, Northwestern Polytechnical University, Xi’an 710072, China; gydebbie@hotmail.com (Y.G.); qiaoxj2009@163.com (X.Q.); loyamuyu@nwpu.edu.cn (X.S.); niuweining@nwpu.edu.cn (W.N.)

**Keywords:** vasoactive intestinal peptide, analogue, recombinant expression, *Escherichia coli*, antimicrobial activity

## Abstract

Antimicrobial peptides represent an emerging category of therapeutic agents with remarkable structural and functional diversity. Modified vasoactive intestinal peptide (VIP) (VIP analogue 8 with amino acid sequence “FTANYTRLRRQLAVRRYLAAILGRR”) without haemolytic activity and cytotoxicity displayed enhanced antimicrobial activities against *Staphylococcus aureus* (*S. aureus*) ATCC 25923 and *Escherichia coli* (*E. coli*) ATCC 25922 than parent VIP even in the presence of 180 mM NaCl or 50 mM MgCl_2_, or in the range of pH 4–10. VIP analogue 8 was expressed as fusion protein thioredoxin (Trx)-VIP8 in *E. coli* BL21(DE) at a yield of 45.67 mg/L. The minimum inhibitory concentration (MIC) of the recombinant VIP analogue 8 against *S. aureus* ATCC 25923 and *E. coli* ATCC 25922 were 2 μM. These findings suggest that VIP analogue 8 is a promising candidate for application as a new and safe antimicrobial agent.

## 1. Introduction

As is known to all, a series of disadvantages brought by the application of classical antibiotics makes it urgent to develop promising and alternative antimicrobial strategies. Recently, intensive research is now invested into identification of new anti-infective therapies [1]. Animals establish a set of innate host defence mechanisms in the process of biological evolution. Antimicrobial peptides represent an emerging category of therapeutic agents with remarkable structural and functional diversity [2]. Many human oral antimicrobial peptides are responsible for playing important roles including maintenance, repairing of oral tissues and defence against oral microbes [3,4]. The role of cationic host-defence peptides in modulating the innate immune response and boosting infection-resolving immunity while dampening harmful pro-inflammatory (septic) responses gives these peptides the potential to become an entirely new therapeutic approach against bacterial infections [5]. Vasoactive intestinal peptide (VIP) is an important signal molecule of the neuroendocrine-immune network [6,7] and plays key roles in a broad spectrum of biological functions including antimicrobial, anti-inflammatory and immune-modulatory activity. Moreover, VIP has been recognized as a host defence peptide [8] and is considered a promising candidate for treatment of several diseases [9]. Furthermore, VIP possesses simple and defined structural properties: small size, cationic charge, and amphipathicity. Moreover, VIP as a kind of brain-gut peptide that belongs to the insulin-pancreatic gastrin-releasing family. Furthermore, VIP usually exhibits strong biological activity at small concentration. Those above advantages make it a promising candidate for application as a new and safe antimicrobials agent.

However, up to now, VIP has not been widely used in agriculture, food or medicine because of the following shortcomings: (1) relatively low antimicrobial activity compared with the classical antibiotics; (2) instability and extreme sensitivity to the peptidases present in body after systemic administration; (3) purification of the natural VIP or artificial synthesis of VIP is low efficient or expensive [10,11]. To overcome those shortcomings, the development of efficacious VIP analogues and an effective, low-cost recombinant expression strategy might promote the VIP-based application. The amino acid sequences and structural properties of peptides significantly affect their action mechanism and selective cytotoxicity [12]. The characteristics of antimicrobial peptides, such as conformation, charge, hydrophobic character, amphipathicity, and second structure, are closely interrelated [13]. Therefore, various approaches such as molecular modification, novel design, and hybrid design were used to improve the biological activities of antimicrobial peptides. VIP derivatives showed the strongest antimicrobial activities against *Escherichia coli* strains that express complete O-antigen-containing LPS [14]. As for the recombinant strategies of antimicrobial peptides, *E. coli* is a widely used expression system because of its unique advantages [15]. Fusion expression strategy can effectively reduce the toxic effect of antimicrobial peptides to the host cells and protect the small antimicrobial peptides from proteolytic degradation. Therefore, recombinant expression of peptides especial antimicrobial peptides needs the help of fusion partner [16]. Thioredoxin (Trx) as a heat-stable and low molecular weight soluble protein in the prokaryotic cytoplasm has been shown to display chaperone-like activity [17].

In the present study, we aimed to design and generate a novel peptide (VIP analogue) with an enhanced antimicrobial potency, low or no-haemolytic activity, and high stability by amino acid substitution based on their physicochemical property and usual antimicrobial mechanism, and establish an effective and low-cost production method by fusion expression of VIP analogue with Trx in *E. coli*. Moreover, we preliminarily investigated the antimicrobial mechanism of VIP analogue by transmission electron microscopy (TEM). In addition, the antimicrobial activities of recombinant VIP analogue were identified.

## 2. Results

### 2.1. Sequence and Structural Properties of VIP and the VIP Analogues

In the current study, we designed seven VIP derivatives (from VIP2 to VIP8) based on the structural properties of VIP and the structure-activity relationship of antimicrobial peptides. The structure and molecular weights of these peptides were verified by electrospray ionization mass spectrometry (ESI-MS). VIP and its analogues had molecular weight values consistent with their theoretical values, suggesting that the peptides were successfully synthesized. The purity of synthesized peptides was above 95%. The chemical modifications achieved to increase the stability of parent VIP resulted in peptides with higher positive charge and (or) hydrophobic nature than VIP (as shown in Table 1). When compared with natural VIP (refer to the VIP1 in Table 1) and the other VIP analogues, VIP 2 and VIP 8 have high cationic charges. After the substitution of Asp^3^, Asp^8^ by Lys^3^, Lys^8^, VIP analogue 2 showed a higher hydrophilicity. The N-terminal truncated VIP derivative has relatively low hydrophilicity compared with other peptides.

### 2.2. Antimicrobial Activity and Possible Mechanism of VIP and VIP Analogue 8

As shown in Table 2, VIP analogue 2, 7 and N-terminal truncated VIP analogue 8 displayed higher antimicrobial activities against *S. aureus* ATCC 25923 and *E. coli* ATCC 25922 than the natural VIP (VIP1). Moreover, VIP did not display antimicrobial activities against *S. aureus* ATCC 25923. However, VIP analogue 2, 7, 8 especially VIP analogue 8 demonstrated high antimicrobial activities against *S. aureus* ATCC 25923 and *E. coli* ATCC 25922.

**Figure 1:**
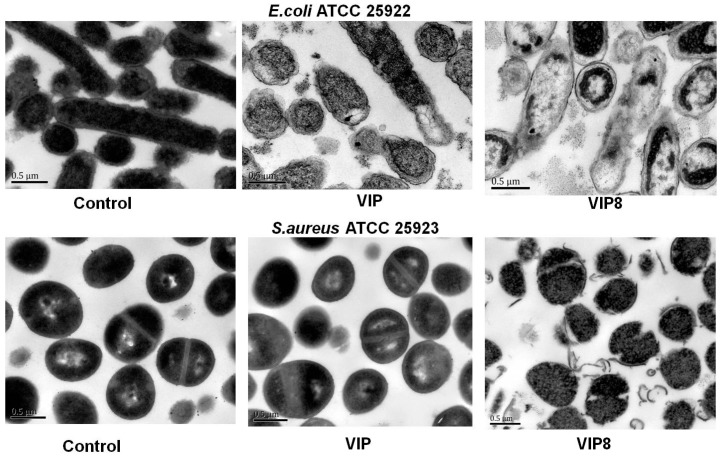
Antimicrobial mechanism of VIP and the VIP analogue 8. Transmission electron micrographs of *E. coli* ATCC 25922; Transmission electron micrographs of *S. aureus* ATCC 25923; bacteria in mid-logarithmic growth were treated with peptides at 1 × MIC for 2 h.

As shown in Figure 1, TEM analysis indicated that the morphology of the bacterial treated with VIP or VIP analogue 8 was altered dramatically. The *E. coli* and *S. aureus* in the control without VIP or VIP analogue treatment present a smooth and integral cell surface and dense internal structure. After treatment with VIP or VIP analogue 8 at the MIC for 3 h, the significant rupture of *E. coli* cell membranes and the release of cellular contents were observed. In comparison with the control, VIP analogue 8 caused significant changes in membrane morphology and intracellular alteration in *S. aureus* cells.

### 2.3. Haemolytic Activity and Cytotoxicity of VIP and VIP Analogue 8

**Figure 2:**
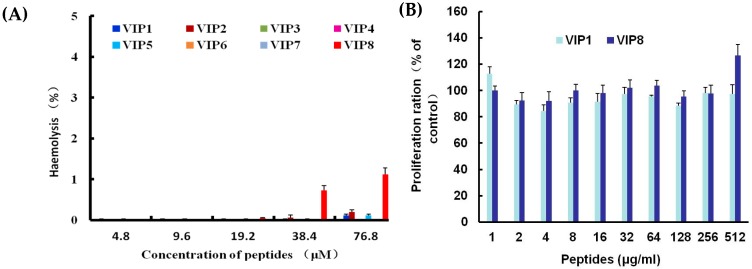

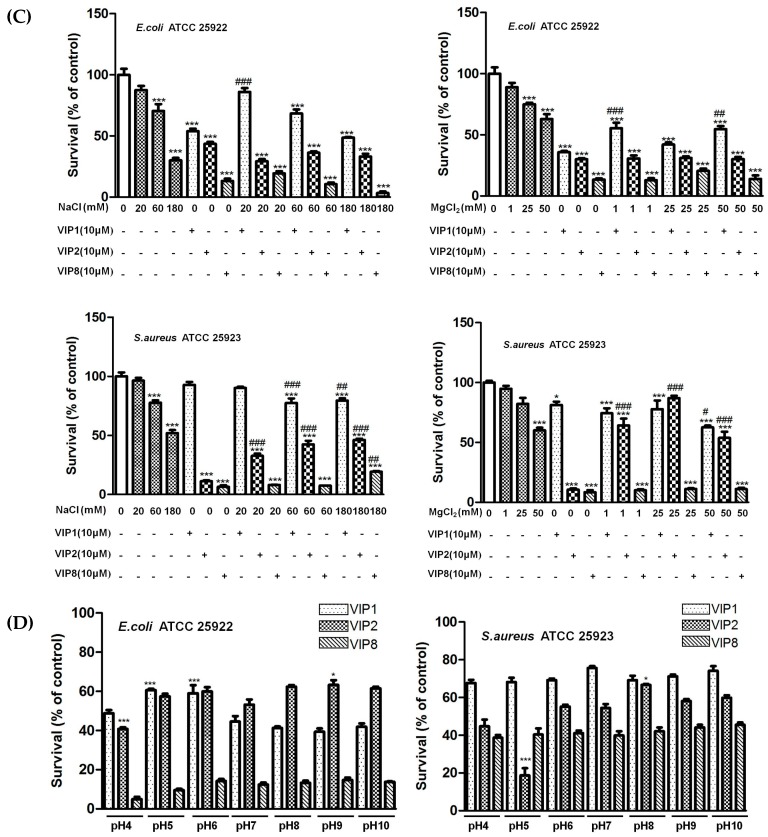
Haemolytic activity of VIP and VIP analogues against pRBCs (**A**), the cytotoxicity of natural VIP (VIP1) and VIP analogue 8 against 3T3-L1 cells (**B**), and effect of the ionic strength (**C**) and pH (**D**) of the medium on the activity of natural VIP (VIP1) and VIP analogue against *E. coli* and *S. aureus*. Bacteria were incubated with VIP1, VIP2, and VIP8 at the concentration of MIC in the absence or presence of different concentrations of NaCl or MgCl_2_ for 3 h. (**C**) * *P* < 0.05 and *** *P* < 0.001 versus peptide-treated bacteria; ^#^ *P* < 0.05, ^##^ *P* < 0.01, and ^###^ *P* < 0.001 versus untreated bacteria at each condition. (**D**) * *P* < 0.05 and *** *P* < 0.001 versus controls at each pH; ^#^ *P* < 0.05, ^##^ *P* < 0.01 and ^###^ *P* < 0.001 versus peptide-treated bacteria at pH 7.0. All data were present as the mean ± SEM (*n* = 4).

In the currently conducted experiments, the haemolytic activity and cytotoxicity of VIP and VIP derives are shown in Figure 2A,B. VIP and VIP derivatives showed no haemolytic activity at the concentrations below 19.2 μM. However, VIP1, 2, 5 and 8 especially VIP8 exhibited slight haemolytic activity (% haemolysis is 1.12%) at high concentration of 76.8 μM. Moreover, VIP8 showed no cytotoxicity at all tested concentrations.

### 2.4. Ionic Strength and pH Influencing Antibacterial Activity of VIP and Its Analogues

As shown in Figure 2C, both VIP analogues 2 and 8 kept their antibacterial activities against *E. coli* ATCC 25922 and *S. aureus* ATCC 25923 at the tested concentrations of NaCl. Moreover, VIP8 displayed high antimicrobial activity against *E. coli* ATCC 25922 and *S. aureus* ATCC 25923 at 50 mM MgCl_2_. However, high concentrations of NaCl and MgCl_2_ significantly inhibited the antibacterial activity of natural VIP. In addition, we observed that the antimicrobial activities of VIP 8 against *E. coli* ATCC 25922 and *S. aureus* ATCC 25923 were not influenced by the medium at the range of pH 4–10 (As shown in Figure 2D).

### 2.5. Expression and Purification of Recombinant VIP Analogue 8

**Figure 3:**
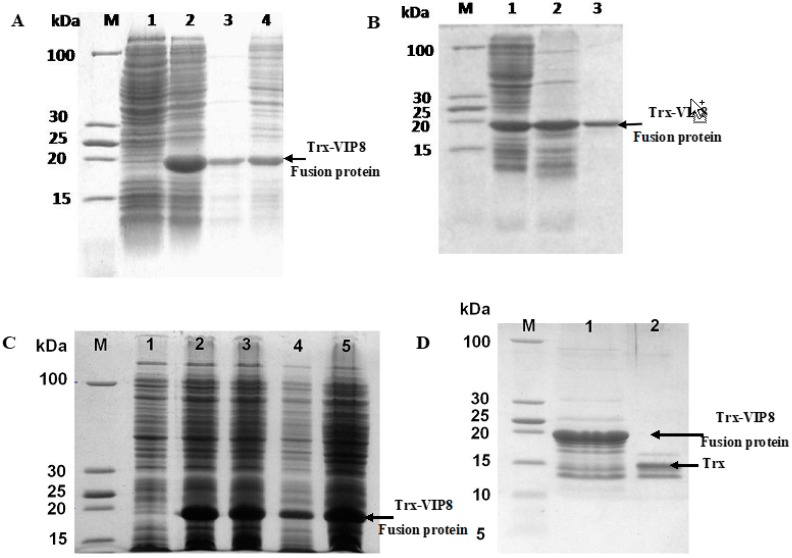
Expression, purification and identification of recombinant VIP analogue 8. (**A**) Expression of pET32a-VIP8 fusion protein analysed by SDS-PAGE. lane M: protein molecular mass marker; lane 1: un-induced BL21(DE3)-pET32a-VIP8; lane 2: induced BL21(DE3)-pET32a-VIP8 by isopropyl-β-d-thiogalactoside; lane 3: supernatant of bacterial lysate; lane 4: precipitation of bacterial lysate; (**B**) Purification of pET32a-VIP8 fusion protein by BeaverBeads^TM^ His-tag Protein Purification kit; lane M: protein molecular mass marker; lane 1: induced whole BL21(DE3)-pET32a-VIP8 protein; lane 2: supernatant of bacterial lysate; and lane 3: purified pET-32a-VIP8 fusion protein; (**C**) Effect of post-induction time on the expression of BL21(DE3)-pET32a-VIP8 protein; lane M: protein molecular marker; lane 1: un-induced BL21(DE3)-pET32a-VIP8; lane 2, 3, 4, 5: induced BL21(DE3)-pET32a-VIP8 by isopropyl-β-d-thiogalactoside for 2, 4, 8, and 20 h respectively; (**D**) A 12% Tricine-SDS-PAGE analysis of the cleavage reaction and purification of the recombinant VIP8. Lane M: low protein molecular weight standard; lane 1: Trx-VIP8 fusion protein without enterokinase digestion; lane 2: purified recombinant VIP8 after enterokinase digestion.

A 113-bp DNA sequence for VIP8 harbouring *Kpn*I and *Xho*I restriction sites was obtained by SOE-PCR. The correct sequence of the clone VIP8 gene and the recombinant fusion vector pET32a-VIP8 were confirmed by agarose gel electrophoresis and DNA sequencing (data not shown). To optimize the expression time, expression levels were analysed at four different times between 2 h and 20 h after induction with 0.4 mM IPTG. As shown in Figure 3, 4 h was optimal for the efficient expression of recombinant proteins. The target fusion protein (Trx-VIP8) was successfully expressed with a 20 kDa-expected band compared to non-induced cells as a negative control. Approximately, 6.85 mg of recombinant VIP8 was recovered from 1 L of fermentation broth with a purity of 85–90%.

### 2.6. Antimicrobial Activity of Recombinant VIP Analogue 8

The antimicrobial activity of recombinant VIP analogue 8 is illustrated by the data presented in Table 3. The MIC of recombinant expressed VIP analogue 8 against *E. coli* ATCC 25922 and *S. aureus* ATCC 25923 was 2 μM. Both 2 μM of recombinant expressed and chemically-synthesized VIP analogue 8 displayed similar inhibitory zone in the agar diffusion test (data not shown), indicating their similar antimicrobial activity. However, their antimicrobial activities were lower than those of classical antibiotics.

## 3. Discussion

The VIP peptide is remarkably well conserved across species and is identical in human, cow, pig, rat, dog, and goat [18]. Even across species, amino acid substitutions are conservative and usually do not result in changes in bioactivity. In mammals, except guinea pigs, the sequence identity is at least 85% [19]. A lines of structure-activity relationship studies of VIP were carried out to identify the portions of peptides involved in interactions with VIP receptors [20]. The N-terminal random coil structure of VIP plays a crucial role in the receptor-selectivity. The presence of α-helical structure forming in 14 amino acid residues between position 10 and 23 in VIP is essential to its biological functions [21]. The current results indicate that some amino residues substitution (Asp^3^→Lys^3^, Asp^8^→Lys^8^) improve the antimicrobial activities of VIP. N-terminal truncated VIP derivative was more potent than natural VIP as an antimicrobial peptide, which was in consistent with previous research [14]. A net positive charge enables antimicrobial peptides electrostatic attraction to the negatively charged microbial membranes [12]. The increase of net positive charge can improve antimicrobial activity within a certain range [22]. However, a higher charge can cause the increase cytoxicity. In the current study, the VIP analogue 2 and analogue 8, although having a very high charge of +7, display no cytotoxicity at all tested concentrations. Hydrophobicity is an important characteristic of antimicrobial peptides, which enables them to penetrate cells and induce membrane lysis [23]. Furthermore, an increase of the hydrophobicity of antimicrobial peptides correlates with its low selectivity and toxicity toward mammalian cells. The contributions of amphipathicity and hydrophobicity to the increase of haemolytic activity have been found in either cyclic peptides or linear unconstrained peptides [24]. However, VIP analogue 8, with a relatively high hydrophobicity, shows slight haemolytic activity at high concentration of 76.8 μM. Moreover, VIP analogues 7 and 8 exhibited strong antimicrobial activities against both *E. coli* ATCC 25922 and *S. aureus* ATCC 25923. However, natural VIP posed no antimicrobial activity against *S. aureus* ATCC 25923. Further experiment indicated that the antimicrobial mechanism was closely related to the disruption of bacterial membranes, which was in consistent with a previous report [14].

The antimicrobial activities of AMPs may be influenced by various factors including salt concentration and pH of the medium. Under low salt concentration, the antibacterial peptide has the best antibacterial activity. From 50 to 125 mmol/L, the activity of human β-defensin 1 (hBD-1) was reduced to 12% rapidly [25]. The antimicrobial activity of human β-defensin 4 (hBD-4) against *E. coli*, *P. aeruginosa*, and *S. aureus* was attenuated in the presence of high concentrations of NaCl [26]. In this study, we found that the high concentration of NaCl inhibited the antimicrobial activities of natural VIP and its analogue 2 against *E. coli* ATCC 25922 and *S. aureus* ATCC 25923, which support the functional relevance of VIP and its analogues in physiological fluids. Previous research indicated that VIP almost completely lost its antimicrobial activity against *E. coli* at the concentration of 150 mM NaCl [27]. However, high concentration of NaCl and MgCl_2_ did not affect the antimicrobial activities of VIP analogue 8. pH changes not only influence the activity of antimicrobial peptides, but they may also affect its secondary structure or amphiphilicity and conformation, etc. The electrostatic interactions between the VIP analogue and bacterial targets may play a critical role in their antimicrobial activity. The net charge of bacterial membrane is more negative when the pH of the medium rises. Therefore, a basic environment could favour stronger interactions of the bacteria with the VIP8 with high cation.

Recombinant DNA technology as a promising strategy for the production of peptide has been widely used to obtain larger quantities of highly bioactive peptides at low cost. The *E. coli* recombinant expression system is suitable for large-scale production with scales of advantages such as easy culture and fast growth [28]. Moreover, in order to avoid toxicity and protelysis of AMPs and increase their expression levels in *E. coli*, several fusion partners have been used to expression AMPs such as Trx, glutathione-S-transferase (GST) and small ubiquitin-like modifier (SUMO) [29,30,31]. In the current study, the soluble fusion protein Trx-VIP8 was successfully expressed in the pET32a(+) expression system with high expression (45.67 mg/L) after 0.4 mM IPTG induction for 4 h at 25 °C. The yield of recombinant purified VIP analogue 8 was about 6.85 mg from 1 L *E. coli* culture. Our strategy provides a method of producing the VIP analogue at a relatively low cost as compared to its chemical synthesis. The recombinant VIP analogue 8 exhibited strong antimicrobial activity against Gram-positive and Gram-negative bacteria investigated in the study.

## 4. Material and Methods

### 4.1. Peptides, Strains, Plasmids, Reagents, and Enzymes

All peptides with purity >95% were synthesized by China Peptides Co., Ltd. (Shanghai, China). The test strains *E. coli* ATCC 25922 and *S. aureus* ATCC 25923 were kindly donated by Prof. Yizhen Wang (Zhejiang University, Hangzhou, China). *E. coli* DH5α, *E. coli* (DE3) pLysS competent cells, restriction enzymes *Kpn*I, *Xho*I, and enterokinase, T4 DNA ligase, DNA ladder and pre-stained protein marker were purchased from Takara (Dalian, China). The pET32a(+) plasmid were purchased from Invitrogen (Beijing, China). Dulbecco’s modified Eagle’s medium (DMEM), penicillin/streptomycin, 0.25% trypsin-ethylenediaminetetraacetic acid (EDTA), and fetal bovine serum (FBS) were purchased from Life Technologies (Grand Island, NY, USA). Other reagents were obtained either from Sangon Chemical Reagent (Shanghai, China) or Sigma (St. Louis, MO, USA). PCR primers were synthesized by Shanghai Sangon Biological Engineering Technology and Services Co., Ltd. (Shanghai, China).

### 4.2. VIP and Its Analogues Design

We designed a series of VIP analogues and replaced some amino acids with alternately arranged hydrophilic amino acids based on the structural characteristics and physicochemical properties analysis, and the antimicrobial mechanism of classic antimicrobial peptides. The amino acid sequences of the VIP and its analogues are listed in Table 1.

### 4.3. Antimicrobial, Hemolytic, Cytotoxicity Assay and Determination of Specific Factors Influencing Antimicrobial Activity

The antimicrobial activities of VIP and its analogues were tested against *S. aureus* ATCC 25923 and *E. coli* ATCC 25922 according to the method reported by Wani et al. [32] with slight modification. The final peptide concentrations ranged from 0.5 to 256 μM. The tests were performed in triplicate using 4 replicates for each experiment. In addition, *S. aureus* ATCC 25923 and *E. coli* ATCC 25922 (2 × 10^6^ CFU/mL) were treated with natural VIP and VIP8 at MIC for 2 h. Samples were prepared according to the previous method [33] and observed with a TEM of Model JEM-1230 (JEOL, Tokyo, Japan) under standard operating conditions. The haemolytic activity of VIP and VIP derivatives were measured using porcine erythrocytes according to previous method [34]. MTT (3-[4,5 dimethylthiozol-2-yl]-2,5-diphenyltetrazolium bromide) was used to determine the cytotoxicity of VIP and its analogue on porcine intestinal epithelial cells IPEC-J2. Stable experiments were conducted with reference to the literature [14] with slightly changes. First, the pH of the mediums was adjusted to acidic (pH 4.0, pH 5.0, and pH 6.0) and basic (pH 8.0, pH 9.0, and pH 10.0) conditions. To investigate the effects of ion strength, the bacterial mediums were supplemented with different concentrations of NaCl (20 mM, 60 mM, and 180 mM) or MgCl_2_ (1 mM, 25 mM, and 50 mM). Then, bacteria were incubated with VIP, VIP analogues 2 and 8, respectively. After 3 h of incubation, bacterial viability was measured with a Synergy HT multimode reader (BioTek, Winooski, VT, USA) at 600 nm.

### 4.4. Construction of Recombinant pET32a-VIP8 Fusion Expression Vector

A 113-bp oligonucleotide of VIP analogue was designed based on *E. coli* codon bias and VIP analogue 8 amino acid sequence. The oligonucleotide sequence of VIP analogue 8 was as follows: 5’-GCTAGGTACCGACGACGACGACAAGTTTACCGCAAATTATACCCGTCTGCGTCGTCAGCTGGCAGTTCGTCGTTATCTGGCAGCAATTCTGGGTCGTCGTTAA***CTCGAG***TCAG-3’. *Kpn*I restriction site (shown in frame), enterokinase cleavage sequence (underlined), and *Xho*I restriction site (italic and bold) with translational termination codons (TAA) were inserted in the N- and C- end of the VIP analogue 8 gene. The VIP analogue 8 gene was amplified by splice overlap extension PCR with three primers (P1: 5’-GCTAGGTACCGACGACGACGACAAGTTTACCGCAAATTATACCCGTCT-3’; P2: 5’-CTGACTCGAGTTAACGACGACCCAGAATTGCTGCCAGATAACGACGAAC-3’; P3: 5’-CCAGATAACGACGAACTGCCAGCTGACGACGCAGACGGGTATAATTTG-3’). The pET32a(+) vector and the PCR products were digested by *Kpn*I and *Xho*I and gel purified; the obtained fragments were ligated to construct the expression vector pET32a-VIP8. The resulting construct was transformed into *E. coli* DH5α and selected on LB plates with ampicillin. The recombinant pET32a-VIP8 plasmid was further confirmed by DNA sequencing.

### 4.5. Expression and Purification of the Recombinant Fusion Protein Trx-VIP8

The recombinant plasmid was transformed into *E. coli* BL21(DE3). A starter culture of BL21-pET32a-Trx-VIP8 was inoculated in 15 mL of LB medium (100 μg/mL ampicillin) with a single colony and incubated at 37 °C with shaking at 250 rpm for overnight. Then, it was inoculated in 500 mL LB medium (100 μg/mL ampicillin). When the density of cells reached OD_600nm_ of 0.6, IPTG was added (final concentration of 0.4 mM). 4 h after induction, the cells were harvested by centrifugation (4000 rpm for 20 min) and resuspended in ice-cold lysis buffer (50 mM NaH_2_PO_4_, 300 mM NaCl, pH 8), and then placed in an ice bath for ultrasonic lysis (200 W, 5 s, 5 s). The supernatant was collected after centrifugation at 12,000× *g* for 10 min at 4 °C, and 30 μL was analysed by sodium dodecyl sulphate polyacrylamide gel electrophoresis (SDS-PAGE). The Trx-VIP8 fusion protein was purified using a BeaverBeads^TM^ His-tag Protein Purification kit according to the manufacturer’s instruction (Beaver Bio., Guangzhou, China), and then desalted with Sephadex G25 medium (GE Healthcare, Uppsala, Sweden) equilibrated with buffer A (50 mM Tris-HCl, pH 8.0, 0.5 mM EDTA, 1 mM DTT, 10% Glycerol). The column was washed with buffer A and the solution corresponding to the elution peak was concentrated with a U-tube concentrator (10 kDa molecular weight cut-off, Merk, Darmstadt, Germany). The protein concentration of the supernatant was determined using a Bradford protein assay kit (KeyGEN Biotech, Nanjing, China).

### 4.6. Enzymatic Cleavage of Trx-VIP8 and Release of VIP8

The purified fusion protein Trx-VIP8 was cleaved by enterokinase (1 μg with respect to 50 μg fusion protein) for 16 h at 25 °C. Then, 5 μL of the cleavage reaction was transferred into Tricine-SDS-PAGE. The recombinant VIP8 was purified using His-tag affinity chromatography, desalted, and separated from enterokinase by Sephadex G25 size exclusion chromatography (GE Healthcare, Little Chalfont, Germany) with ddH_2_O as an elution buffer. The released and purified VIP8 was lyophilized by freeze-drying and further used for electrospray ionization mass spectrometry (ESI-MS), antimicrobial and anti-inflammatory activities assays.

### 4.7. Antimicrobial Activity Assay of Recombinant VIP8

Both the agar diffusion test and modified broth microdilution method were used to evaluate the antimicrobial activity of recombinant expressed VIP8. For the Oxford cup agar diffusion, the sample was added to an Oxford cup, which was then placed on a Mueller-Hinton agar plate containing 1 × 10^5^ colony-forming units of *S. aureus* ATCC 25922 and *E. coli* ATCC 25923. Chemically-synthesized VIP8 and ampicillin were used as the positive control. The minimal inhibition concentration was determined by the method reported by Wani et al. [32].

### 4.8. Statistical Analysis

Data were analysed by one-way analysis of variance (ANOVA) and by using Student’s *t* test (SPSS 19.0, Chicago, IL, USA). All data were shown as mean ± standard error of mean (SEM), and differences were considered to be statistically significant at *P* < 0.05.

## 5. Conclusions

In summary, this study screened two VIP analogues without cytotoxicity and haemolysis activity, and with enhanced antimicrobial activities over the parent VIP, and provided a simple, low cost strategy for producing the neuro-immunomodulatory peptide-VIP analogue without affecting its antimicrobial activity and anti-inflammatory activity. These results suggest that it may be feasible and promising to develop VIP analogue as a potent antimicrobial agent for food or animal breeding industry.

## Figures and Tables

**Table 1:** Sequences and structural properties of VIP and the VIP analogues.

Name	Amino Acides Sequence	Charge	GRAVY ^a^	Molecular Weight
VIP1 ^b^	HSDAVFTDNYTRLRKQMAVKKYLNSILN	+3	−0.639	3326.8
VIP2	HSKAVFTKNYTRLRKQMAVKKYLNSILN	+7	−0.668	3352.9
VIP3	HSDAVFTDNSTRLRKQMAVKKSLNSILN	+3	−0.604	3174.6
VIP4	HSDAVFTDNYTRLRKQMAVKKYLNSILT	+3	−0.539	3313.8
VIP5	HSKAVFTKNYTRLRKQMAVKKYLNSILT	+7	−0.568	3339.9
VIP6	HSDAVFTDNSTRLRKQMAVKKSLNSILT	+3	−0.504	3161.6
VIP7 ^c^	HSDAVFTANYTRLRRQLAVRRYLAAILGRR	+6	−0.350	3545.2
VIP8 ^c^	FTANYTRLRRQLAVRRYLAAILGRR	+7	−0.360	3035.6

^a^ Grand average of hydropathicity; ^b^ VIP1 is the natural vasoactive intestinal peptide; ^c^ VIP7 and VIP8 are cited from [14].

**Table 2:** Antimicrobial activity of VIP and its analogues. Microorganisms used were *E*. *coli* ATCC 25922 and *S*. *aureus* ATCC 25923. Cecropin P1 and blank (virgin media) were used as positive and negative controls.

Pathogen	*E*. *coli* ATCC 25922		*S*. *aureus* ATCC 25923	
Compound	MIC ^a^	MBC ^b^	MIC	MBC
VIP1 ^c^	64	128	>256	>256
VIP2	16	32	64	128
VIP3	>256	>256	>256	>256
VIP4	>256	>256	>256	>256
VIP5	>256	>256	>256	>256
VIP6	>256	>256	>256	>256
VIP7	8	8	16	32
VIP8	2	4	2	4
Cecropin P1	2	4	8	8

^a^ MIC means minimum inhibitory concentration in μM; ^b^ MBC means minimum bactericidal concentration in μM; ^c^ VIP1 is the natural vasoactive intestinal peptide.

**Table 3:** Antimicrobial activities of recombinant VIP8 ^a^.

Microorganism		Cecropin P1	Natural VIP	Recombinant VIP8
		MIC (μM)	MIC (μM)	MIC (μM)
G+	*S. aureus* ATCC 25923	8	>256	2
G−	*E. coli* ATCC 25922	2	64	2

^a^ MIC means minimal inhibitory concentration.
